# Supplementary material for: Mechanisms governing the pioneering and redistribution capabilities of the non-classical pioneer PU.1
Source: Nat Commun. 2020 Jan 21;11:402. doi: 10.1038/s41467-019-13960-2 (PMC6972792; doi:10.1038/s41467-019-13960-2)
Supplement: Supplementary file 7 — Source data [file 41467_2019_13960_MOESM7_ESM.zip › Source_Data/FigureS3/FigureS3J_MotifScanOutput/homerResults/motif11.similar.html]

motif11

## Information for motif11

A
C
G
T
A
C
G
T
C
T
A
G
G
A
T
C
C
T
A
G
G
A
T
C
G
T
C
A
C
G
T
A
A
G
C
T
  
Reverse Opposite:  

T
C
G
A
A
C
G
T
A
C
G
T
C
T
A
G
G
A
T
C
C
T
A
G
G
A
T
C
C
G
T
A
C
G
T
A
  

|  |  |
| --- | --- |
| p-value: | 1e-127 |
| log p-value: | -2.928e+02 |
| Information Content per bp: | 1.714 |
| Number of Target Sequences with motif | 1896.0 |
| Percentage of Target Sequences with motif | 14.37% |
| Number of Background Sequences with motif | 2580.2 |
| Percentage of Background Sequences with motif | 7.07% |
| Average Position of motif in Targets | 388.2 +/- 327.8bp |
| Average Position of motif in Background | 324.4 +/- 198.8bp |
| Strand Bias (log2 ratio + to - strand density) | 0.1 |
| Multiplicity (# of sites on avg that occur together) | 1.10 |
| Motif File: | file (matrix) reverse opposite |

### Similar de novo motifs found

|  |  |  |  |  |  |  |  |
| --- | --- | --- | --- | --- | --- | --- | --- |
| Rank | Match Score | Redundant Motif | P-value | log P-value | % of Targets | % of Background | Motif file |
| 1 | 0.773 | T C A G G A C T A C G T C A T G C G A T A C T G G A C T G T C A T C G A C A G T G A T C G C T A | 1e-123 | -284.245186 | 18.53% | 10.30% | motif file (matrix) |
| 2 | 0.850 | T C A G A G C T A C G T C G T A A G T C C T A G G A T C G T C A C T G A G C A T T A G C | 1e-122 | -282.253288 | 24.29% | 14.95% | motif file (matrix) |
| 3 | 0.954 | G C A T A C G T C T A G A G T C C A T G G T A C C T G A T G C A | 1e-106 | -245.974832 | 16.21% | 8.99% | motif file (matrix) |
| 4 | 0.913 | T C G A A C G T A C G T C A T G A G T C C T G A G C A T G T C A C G T A A G T C | 1e-90 | -207.307899 | 28.96% | 20.24% | motif file (matrix) |
| 5 | 0.758 | T A C G G C A T C A G T C T G A T A G C A C T G G A T C G T C A T C G A C G A T T A G C G C T A | 1e-81 | -186.742874 | 20.00% | 12.92% | motif file (matrix) |
